# Amplified Spontaneous Emission Threshold Dependence on Determination Method in Dye-Doped Polymer and Lead Halide Perovskite Waveguides

**DOI:** 10.3390/molecules27134261

**Published:** 2022-07-01

**Authors:** Stefania Milanese, Maria Luisa De Giorgi, Luis Cerdán, Maria-Grazia La-Placa, Nur Fadilah Jamaludin, Annalisa Bruno, Henk J. Bolink, Maksym V. Kovalenko, Marco Anni

**Affiliations:** 1 Dipartimento di Matematica e Fisica “Ennio De Giorgi”, Università del Salento, Via per Arnesano, 73100 Lecce, Italy; marialuisa.degiorgi@unisalento.it (M.L.D.G.); marco.anni@unisalento.it (M.A.); 2 Instituto de Ciencia Molecular, Universidad de Valencia, 46980 Paterna, Spain; lcerdanphd@gmail.com (L.C.); mg.laplaca@uv.es (M.-G.L.-P.); henk.bolink@uv.es (H.J.B.); 3 Energy Research Institute @ NTU (ERI@N), Nanyang Technological University, Singapore 637553, Singapore; nurf0032@e.ntu.edu.sg (N.F.J.); annalisa@ntu.edu.sg (A.B.); 4 Department of Chemistry and Applied Biosciences, Institute of Inorganic Chemistry, ETH Zürich, CH-8093 Zürich, Switzerland; mvkovalenko@ethz.ch; 5 Laboratory for Thin Films and Photovoltaics, Empa—Swiss Federal Laboratories for Materials Science and Technology, CH-8600 Dübendorf, Switzerland

**Keywords:** dye doped waveguides, lead halide perovskites, nanocrystals, amplified spontaneous emission, optical gain, laser

## Abstract

Nowadays, the search for novel active materials for laser devices is proceeding faster and faster thanks to the development of innovative materials able to combine excellent stimulated emission properties with low-cost synthesis and processing techniques. In this context, amplified spontaneous emission (ASE) properties are typically investigated to characterize the potentiality of a novel material for lasers, and a low ASE threshold is used as the key parameter to select the best candidate. However, several different methods are currently used to define the ASE threshold, hindering meaningful comparisons among various materials. In this work, we quantitatively investigate the ASE threshold dependence on the method used to determine it in thin films of dye-polymer blends and lead halide perovskites. We observe a systematic ASE threshold dependence on the method for all the different tested materials, and demonstrate that the best method choice depends on the kind of information one wants to extract. In particular, the methods that provide the lowest ASE threshold values are able to detect the excitation regime of early-stage ASE, whereas methods that are mostly spread in the literature return higher thresholds, detecting the excitation regime in which ASE becomes the dominant process in the sample emission. Finally, we propose a standard procedure to properly characterize the ASE threshold, in order to allow comparisons between different materials.

## 1. Introduction

The first demonstration of lasing from organic dyes in solution in 1966 [1,2] encouraged researches toward the realization of organic semiconductor light-emitting sources [3,4,5,6]. In particular, due to the easy modulation of emission properties through chemical tailoring, the high efficiency, and the low cost of raw materials and processing, several active compounds have been synthesized, such as dyes, conjugated polymers, organic crystals, and dendritic starburst molecules [3,7]. Significant advancements toward the realization of tunable solid-state dye lasers have been made thanks to the incorporation of small molecules in polymeric waveguides. In this regard, in order to improve the operational stability of devices and reduce the thermal and chemical photodegradation of dyes, numerous studies have carried out focusing both on the choice of the active organic dye and the embedding polymeric structure [8,9,10,11,12,13].

More recently, another class of active materials has drawn increasing attention: lead halide perovskites, which initially were investigated for their possible use as active materials in photovoltaic devices [14,15,16,17]. After the first demonstration of high optical gain in solution-processed hybrid organic–inorganic halide perovskites [18], they also captured the attention of the scientific community as possible active materials for laser devices [15,19,20,21,22]. Following the demonstration of stimulated emission both in hybrid and fully inorganic perovskites [23,24,25,26,27], several optically pumped lasers have been realized [28,29,30,31,32,33,34].

A common step in the characterization of a novel active material for laser applications is the analysis of its Amplified Spontaneous Emission (ASE) properties, which can be performed without any resonator providing optical feedback. The active material is deposited in form of a thin film in order to obtain a planar asymmetric waveguide and the ASE is observed by applying the variable pump intensity (VPI) method, i.e., by acquiring the photoluminescence (PL) spectra as a function of the excitation energy density. At low excitation densities, the PL spectra show only spontaneous emission, while, for excitation densities that are high enough, the ASE band starts to be visible, inducing a line shape variation, a spectral narrowing, and a stronger increase of the output intensity.

The ability of a material to amplify radiation is typically quantified by an ASE threshold, with the sample with the lowest threshold considered the most suitable for the realization of a laser device. However, the ASE threshold does not have a unique definition and several methods have been adopted to determine it, making comparison between different values extremely difficult. A first step in the investigation of the ASE threshold dependence on the determination method was performed on polymeric active waveguides [35], evidencing differences of up to one order of magnitude between the threshold values obtained with different methods for the same film.

To investigate the generality of this result, in this work, we study the ASE threshold dependence on its determination method for a total of seven thin films of lead halide perovskites of different dimensionality, and organic dyes embedded in polymeric matrices. In particular, we determine the ASE threshold of four different perovskite samples, with different dimensionality and chemical composition (fully inorganic or hybrid organic-inorganic), namely, ${\mathrm{CsPbBr}}_{3}$ nanocrystals (NCs) thin films (with and without hexamethyldisilazane (HMDS)-functionalized substrate) [36], a polycrystalline ${\mathrm{MAPbBr}}_{3}$ thin film [37], and a quasi-2D ${\mathrm{BA}}_{3}{\mathrm{MA}}_{3}{\mathrm{Pb}}_{5}{\mathrm{Br}}_{16}$ perovskite film [26]. For the dye-polymer blends, we analyze the ASE properties of poly(methyl methacrylate) (PMMA) film doped with perylene orange (PO) [38] and two poly(2-hydroxyethyl methacrylate) (pHEMA) thin films doped with sulforhodamineB (SRhB) [39], and rosamine4 [40], respectively, taken as prototype materials of the dye-polymer blends class. For each sample, the ASE threshold is determined through the application of seven different methods (see below), demonstrating a systematic value dependence on the method, despite the very different nature of the active layer. We also demonstrate that the methods leading to the smallest threshold values allow the determination of the excitation regime in which the ASE appears, while the methods most spread in the literature lead to higher values, corresponding to the excitation regime in which the ASE starts to dominate the emission. The relative difference among the various methods strongly depends on the ASE efficiency, and can vary from 30% in materials with strongly growing ASE (such as most lead halide perovskite films) up to six times more in dye-doped polymer films. Overall, our results demonstrate that the comparison of different materials on the basis of the ASE threshold obtained with different methods is, in general, not meaningful. In this sense, a correct knowledge of the kind of information that each method determines is fundamental to properly relate the threshold values to the ASE properties of a given material. Our results allow us to propose a standard procedure for correctly characterizing the ASE threshold, allowing a meaningful comparison between different materials.

## 2. Results

### 2.1. State of Art

As a first step, deep research on the state of the art was undertaken. In particular, we performed a Scopus search looking for all papers containing the following strings of words, “Amplified–Spontaneous–Emission–Dye–Film” and “Amplified–Spontaneous–Emission–Perovskite”, through a “Title–Abstract–Key" search type. In this way, we selected all the papers dealing with ASE in dye-doped and perovskite films, excluding all the papers on solutions or lasers. We found and analyzed 112 and 129 papers for dye-polymer blends and perovskites, respectively, evidencing the existence of several different methods to determine the ASE threshold. In particular, these include:

- **No threshold**: papers that do not provide any estimate of the ASE threshold [41,42,43];
- **Qualitative**: papers that provide an ASE threshold value, but do not specify how they calculate it [44,45];
- **Slope general**: the ASE threshold is defined as the point of slope change in the plot of the emission intensity as a function of the excitation density, without specifying what type of emission intensity is detected [46,47,48,49];
- **Slope total integrated**: the slope variation is determined from the input–output plot of the total intensity, integrated across the sample emission spectral range [50,51,52,53];
- **Slope peak**: the slope variation is determined from the input–output plot of the intensity at the ASE peak wavelength [26,54,55,56];
- **Slope ASE integrated**: the slope variation is determined from the input–output plot of the spectrally integrated contribution of the ASE emission, separated from the spontaneous emission peak [24,57];
- **FWHM/2**: the ASE threshold is defined as the excitation density at which the emission full width at half maximum (FWHM) becomes one half of the FWHM value obtained at low excitation density, corresponding to the spontaneous emission value [58,59];
- **FWHM narrowing**: the ASE threshold is defined as the excitation density that corresponds to the beginning of the narrowing of the line [60,61];
- **FWHM crossing**: the ASE threshold is determined as the excitation density of the crossing point between the two best-fit lines describing the constant FWHM at low excitation density and the FWHM narrowing regime [62,63,64];
- **Visual**: the ASE threshold is defined as the excitation density at which the spectral lineshape starts to change due to the appearance of an early-stage ASE band [37,65,66,67];
- **Gain**: the ASE threshold is defined as the excitation density at which the net gain of the material becomes zero [68];
- **Numerical**: the ASE threshold is determined numerically starting from an analytical expression that describes the increase of ASE intensity with the excitation density [69].

**Figure 1:**
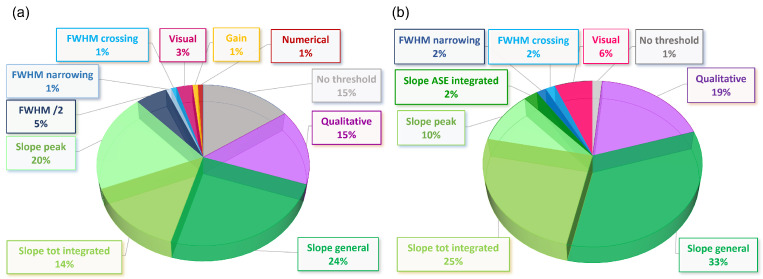
Pie charts showing the percentage distribution of the methods used to define the ASE threshold in (**a**) dye-polymer blends and (**b**) perovskites.

By looking at the percentage distribution of the different methods (see Figure 1), it can be observed that about 15% of the papers on ASE in dye-doped waveguides do not provide any ASE threshold, even if ASE is central in the article discussion. Meanwhile, that percentage goes down to only 1% of the papers on ASE in lead halide perovskites. Considering that the most recent paper without an ASE estimate in dye-polymer samples dates back to 2013, this difference evidences increased attention on quantitative ASE investigation in novel active materials. In addition, about 15–20% of the papers are just qualitative, as a threshold value is present, but without a clear explanation of the method used to determine it. Concerning the distributions of other methods, many similarities are observed. In particular, for both classes of materials, the ASE threshold is mainly determined by methods based on the slope change in the input–output intensity plot, reaching a total of 58% and 70% for dyes and perovskites, respectively (green slices in pie charts, Figure 1). On the contrary, the methods based on the FWHM excitation density dependence are much less spread, being used in 4–7% of the papers, and a similar percentage is also found for the visual method. In the following, we will thus focus our attention on the methods that are mostly spread in the literature, namely: slope total integrated (hereafter, $I_{\mathrm{TOT}}$), slope peak ($I_{\mathrm{peak}}$), slope ASE integrated ($I_{\mathrm{ASE}}$), FWHM/2, FWHM crossing (${\mathrm{FWHM}}_{\mathrm{cross}}$), FWHM narrowing (${\mathrm{FWHM}}_{\mathrm{narr}}$), and visual.

### 2.2. ASE in Lead Halide Perovskites

**Figure 2:**
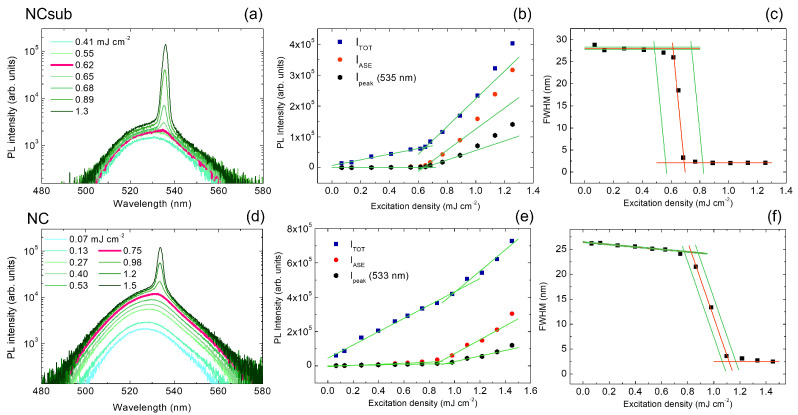
(**a**–**d**) Excitation density dependence of the PL spectra. The thicker pink line evidences the first spectrum in which the lineshape is modified by the ASE presence. (**b**–**e**) Excitation density dependence of the total integrated intensity ($I_{\mathrm{TOT}}$), the ASE integrated intensity ($I_{\mathrm{ASE}}$) and ASE peak intensity ($I_{\mathrm{peak}}$). Green lines represent the best-fit curves. (**c**–**f**) Excitation density dependence of the spectral linewidth (FWHM). The red lines are the best-fit curves and the green lines are the limits of the uncertainty range. The top and bottom rows show the results for NCsub and NC, respectively.

As first samples, we investigated two fully inorganic ${\mathrm{CsPbBr}}_{3}$ NCs perovskite thin films, with (NCsub, Figure 2a–c) and without (NC, Figure 2d–f) HMDS-functionalization of the substrate [36].

The excitation density dependence of the PL spectra of the NCsub sample shows a spontaneous emission band centered at about 530 nm at excitation densities below $0.6\;\mathrm{mJ}\;{\mathrm{cm}}^{-2}$, while, for higher excitation densities, a narrow ASE band centered at about 535 nm becomes visible (see Figure 2a). The lowest excitation density that allows the observation of a variation of the PL spectrum lineshape (visual ASE threshold) is $0.62\;\mathrm{mJ}\;{\mathrm{cm}}^{-2}$ (pink thicker line evidenced in Figure 2a). The ASE threshold was determined with the most used methods in literature for perovskite materials, based on the slope variation in the excitation density dependence of the emitted light intensity, namely $I_{\mathrm{TOT}}$, $I_{\mathrm{ASE}}$, and $I_{\mathrm{peak}}$ (see Figure 2b), obtaining $\left(0.652\pm 0.025\right)\;\mathrm{mJ}\;{\mathrm{cm}}^{-2}$, $\left(0.643\pm 0.019\right)\;\mathrm{mJ}\;{\mathrm{cm}}^{-2}$, and $\left(0.651\pm 0.030\right)\;\mathrm{mJ}\;{\mathrm{cm}}^{-2}$, respectively. Moreover, the typical ASE-induced line narrowing can be observed as the excitation density increases (see Figure 2c). The excitation density at which the line narrowing begins is $\left(0.48\pm 0.04\right)\;\mathrm{mJ}\;{\mathrm{cm}}^{-2}$ (application of the ${\mathrm{FWHM}}_{\mathrm{narr}}$ ASE threshold). Finally, the excitation density of the crossing of the two best-fit lines over the knee of the curve (${\mathrm{FWHM}}_{\mathrm{cross}}$ threshold) and the one at which the FWHM reaches one half of the initial value (FWHM/2 threshold) are $\left(0.614\pm 0.074\right)\mathrm{mJ}\;{\mathrm{cm}}^{-2}$ and $\left(0.656\pm 0.075\right)\mathrm{mJ}\;{\mathrm{cm}}^{-2}$, respectively. All the ASE thresholds are also reported in Table 1.

We then performed a similar analysis for the NC sample. The PL spectra (see Figure 2d) show the spontaneous emission band for excitation densities up to $0.53\;\mathrm{mJ}\;{\mathrm{cm}}^{-2}$, with the evidence of ASE appearance at an excitation density of $0.75\;\mathrm{mJ}\;{\mathrm{cm}}^{-2}$. Figure 2e provides the analysis of the total integrated, ASE integrated, and ASE peak intensity, which results in the following ASE threshold values: $\left(1.01\pm 0.18\right)\;\mathrm{mJ}\;{\mathrm{cm}}^{-2}$ ($I_{\mathrm{TOT}}$), $\left(0.884\pm 0.081\right)\;\mathrm{mJ}\;{\mathrm{cm}}^{-2}$ ($I_{\mathrm{ASE}}$), and $\left(0.931\pm 0.078\right)\;\mathrm{mJ}\;{\mathrm{cm}}^{-2}$ ($I_{\mathrm{peak}}$). Finally, we investigated the excitation density dependence of the FWHM (see Figure 2f), which showed an initial weak narrowing, even in the excitation regime in which only spontaneous emission is present ($0.1-0.7\;\mathrm{mJ}\;{\mathrm{cm}}^{-2}$), followed by a stronger narrowing due to the ASE appearance. We thus modified the FWHM fitting procedure, replacing the initial constant function with a decreasing linear function and obtaining as ASE thresholds $\left(0.688\pm 0.033\right)\;\mathrm{mJ}\;{\mathrm{cm}}^{-2}$ for ${\mathrm{FWHM}}_{\mathrm{narr}}$, $\left(0.827\pm 0.032\right)\;\mathrm{mJ}\;{\mathrm{cm}}^{-2}$ for ${\mathrm{FWHM}}_{\mathrm{cross}}$, and $\left(0.977\pm 0.030\right)\;\mathrm{mJ}\;{\mathrm{cm}}^{-2}$ for FWHM/2. The results obtained from the application of all the methods for the NC sample are summarized in Table 1.

We then performed the same analysis starting from the excitation density dependence of the PL spectra of the quasi-2D and ${\mathrm{MAPbBr}}_{3}$ (MAPB) samples (see Figures S1 and S2 and the results description in the Supporting Information), obtaining the ASE threshold values reported in Table 1. We can notice the lack of the $I_{\mathrm{TOT}}$ threshold for the quasi-2D sample; the absence of a slope variation in the excitation density dependence of the total emission indeed prevents the determination of the ASE threshold with the $I_{\mathrm{TOT}}$ method (see Figure S5). The high contribution of the spontaneous emission to the whole emission above the ASE threshold overshadows the contribution of the stimulated emission and prevents the presence of an inflection point (see Figure S5).

### 2.3. ASE in Dye-Polymer Blends

**Figure 3:**
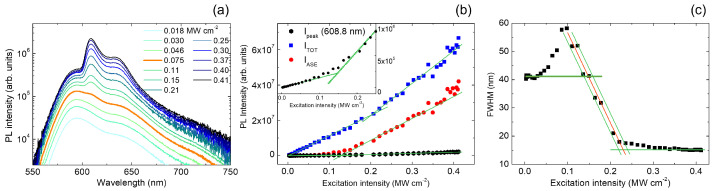
(**a**) Excitation intensity dependence for a selection of the PL spectra of the SRhB sample. The thicker orange line evidences the first spectrum in which the lineshape is modified by the ASE presence. (**b**) Excitation intensity dependence of the total integrated intensity ($I_{\mathrm{TOT}}$, black dots), ASE integrated intensity ($I_{\mathrm{ASE}}$, red dots), and of the intensity at the ASE band peak wavelength ($I_{\mathrm{peak}}$, blue dots). Inset: zoom of the $I_{\mathrm{peak}}$ plot at the slope change. The green lines are the best-fit curves. (**c**) Excitation intensity dependence of the PL spectra FWHM. The red lines are the best-fit curves and the green lines are the limits of the uncertainty range.

The PL spectra of SRhB dye embedded in a pHEMA matrix as a function of the excitation intensity [39] across the ASE threshold are reported in Figure 3a. At low excitation intensity, the spontaneous emission spectrum is characterized by the presence of two spectral bands, centered at about 590 nm and 635 nm, while when increasing the excitation intensity, a new band centered at about 609 nm begins to be visible starting from $0.075\;\mathrm{MW}\;{\mathrm{cm}}^{-2}$, corresponding to the visual threshold [70].

The analysis of the total integrated intensity, the ASE integrated intensity and the ASE peak intensity is provided in Figure 3b, and the respective ASE thresholds are (0.202$\pm 0.018)\;\mathrm{MW}\;{\mathrm{cm}}^{-2}$ for $I_{\mathrm{TOT}}$, $\left(0.1292\pm 0.0056\right)\;\mathrm{MW}\;{\mathrm{cm}}^{-2}$ for $I_{\mathrm{ASE}}$, and (0.149 ± 0.010) MW $\;{\mathrm{cm}}^{-2}$ for $I_{\mathrm{peak}}$.

Finally, starting from the photoluminescence measurements, we also investigated the dependence of the spectral linewidth on the excitation intensity (see Figure 3c). In this sample, we observe an unusual FWHM dependence on the excitation regime. In particular, the FWHM is initially constant around 40 nm (up to about $0.03\;\mathrm{MW}\;{\mathrm{cm}}^{-2}$); then, it increases up to 60 nm at $0.1\;\mathrm{MW}\;{\mathrm{cm}}^{-2}$ and finally decreases until reaching a minimum plateau at about 15 nm for higher excitation intensities. This peculiar behavior, ascribed to the presence of SRhB dimers and higher order aggregates [39], can be understood by observing that at low pump intensity, the FWHM is determined by the 590 nm peak linewidth. As the pump intensity increases, the sample shows not only the appearance of the ASE band around 609 nm, but also a clear increase of the relative intensity of the shoulder at 635 nm. The FWHM increase in the range $\left(0.03–0.1\right)\;\mathrm{MW}\;{\mathrm{cm}}^{-2}$ is thus due to the increasing contribution of the 635 nm shoulder to the total line width while, at even higher pump intensities, the ASE peak at 609 nm progressively dominates the emission, giving rise to the line narrowing.

In order to take into account this behavior, we adapted the ${\mathrm{FWHM}}_{\mathrm{narr}}$ method, considering that the point of beginning of the line narrowing usually represents the point at which the ASE appearance modifies the FWHM with respect to the spontaneous emission method. As in this sample the ASE appearance initially increases the FWHM, we replaced the ${\mathrm{FWHM}}_{\mathrm{narr}}$ method with a ${\mathrm{FWHM}}_{\mathrm{dev}}$ one, in which the ASE threshold is determined as the excitation density at which the linewidth starts to exceed the initial constant value ((0.0337±0.0022)$\mathrm{MW}\;{\mathrm{cm}}^{-2}$). For the ${\mathrm{FWHM}}_{\mathrm{cross}}$, we maintained the definition of the parameter and calculated the threshold as the crossing between the extension of the first constant fit line up to $0.2\;\mathrm{MW}\;{\mathrm{cm}}^{-2}$ and the linear decreasing one, resulting in $\left(0.1485\pm 0.0068\right)\;\mathrm{MW}\;{\mathrm{cm}}^{-2}$. Finally, we took into account the best-fit parameter obtained from the constant fit at low excitation density (∼41nm) as the reference linewidth value for the calculation of the ASE threshold through the FWHM/2 method ($\left(0.2128\pm 0.0060\right)\;\mathrm{MW}\;{\mathrm{cm}}^{-2}$). The values obtained from the application of the various methods for SRhB sample are summarized in Table 2.

We then performed a similar analysis on rosamine4 and perylene orange samples, whose spectral features and results descriptions are reported in Figures S3 and S4. Similarly to what is observed for quasi-2D sample, in Table 2, the experimental data corresponding to the $I_{\mathrm{TOT}}$ thresholds are absent; rosamine4 and PO plots of the total integrated intensity as a function of the excitation intensity do not show the evident slope change needed for the determination of the ASE threshold (see Figure S5).

Table 2 summarizes all the ASE threshold values obtained for the three dye-polymer blends.

## 3. Discussion

**Figure 4:**
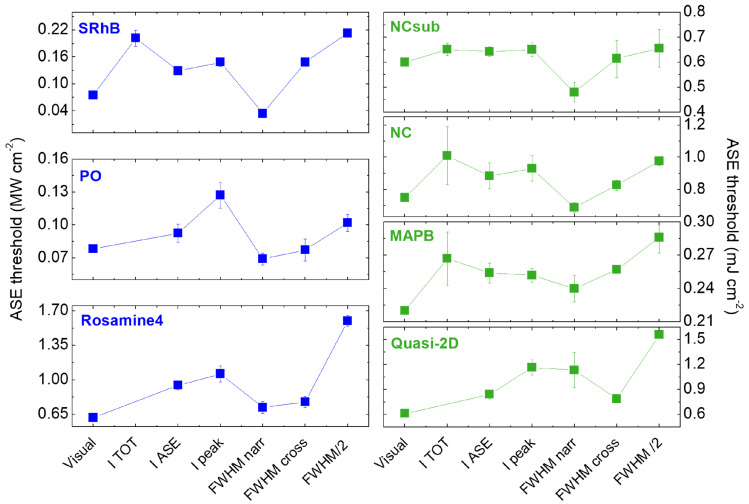
ASE threshold values for SRhB, PO, and rosamine4 (on the **left**); NCsub, NC, MAPB and quasi-2D (on the **right**), as a function of the adopted method.

A complete overview of the method dependence of ASE thresholds for all the investigated samples is provided by Figure 4, in which all the obtained values for all samples are plotted. Even if the investigated samples are very different, it is interesting to observe some common features.

First of all, in all the samples the lowest value is obtained either from the application of the visual or the ${\mathrm{FWHM}}_{\mathrm{narr}}$ method. On the other side, the three different methods that involve the input–output intensity plots always lead to comparable thresholds within each other (within less than 30%), but are up to six times larger than the minimum values (for SRhB). These values are also typically comparable with the threshold obtained with the FWHM/2 method, with the exceptions of rosamine4 and quasi-2D perovskite, which are characterized by a smooth FWHM narrowing.

Moving to a deeper analysis of the results obtained from the three methods exploiting the input–output intensity plots, we evidence that for three samples (namely PO, rosamine4, and quasi-2D perovskite), it is not possible to find a slope change from the $I_{\mathrm{TOT}}$ plot and therefore extract an ASE threshold through one of the methods most frequently used in the literature (see Figure S5). This result can be ascribed to the high relative contribution of the spontaneous emission to the total emission even at high pump densities, which masks the contribution of the stimulated emission and prevents the presence of the inflection point. This attribution is also consistent with the results of Li et al [71], which report a decrease of the ASE contribution to the total emitted PL passing from a 3D to a quasi-2D structure of the ${\mathrm{CsPbBr}}_{3}$ perovskite; this resulted in the lack of the inflection point in the $I_{\mathrm{TOT}}$ plot, thus preventing the determination of the threshold.

The importance of the contribution of the spontaneous emission to the analyzed intensity in the determination of the ASE threshold is also confirmed by the values obtained by the application of the other two output intensity-based methods: $I_{\mathrm{ASE}}$ and $I_{\mathrm{peak}}$. Even if the two methods typically provide similar threshold values, the $I_{\mathrm{ASE}}$ threshold is systematically lower than the $I_{\mathrm{peak}}$ one, evidencing the greater capability of this method to probe the ASE appearance. Considering that the $I_{\mathrm{ASE}}$ method is more time-consuming, since it needs to separate the ASE and the spontaneous emission contributions from the total area of the spectra, and that the values provided by the $I_{\mathrm{peak}}$ method are very similar, we suggest $I_{\mathrm{peak}}$ as the method of best compromise between reliable ASE threshold and ease of application.

The general similarity between the values obtained from the intensity plots and from the FWHM/2 method, and the evidence that the ${\mathrm{FWHM}}_{\mathrm{narr}}$ and visual methods always provide the lowest threshold values suggest that the different methods are able to evidence the presence of ASE in different excitation regimes.

**Figure 5:**
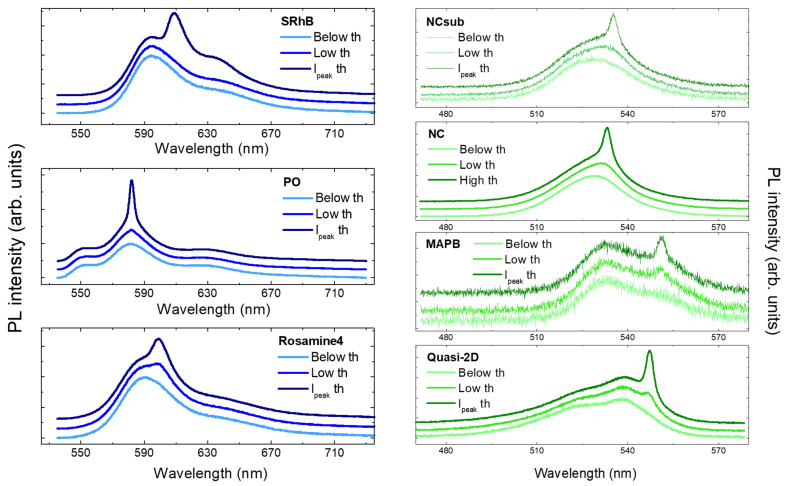
Comparison of PL spectra corresponding to different ASE thresholds. In particular, the belowth spectrum corresponds to PL acquired at low excitation density, below threshold; the lowth plot corresponds to a pump value closest to the minimum ASE threshold found for each sample; finally, the $I_{\mathrm{peak}}$th curve is the PL plot recorded at the excitation density closest to the $I_{\mathrm{peak}}$ ASE threshold (the excitation density of the Lowth and $I_{\mathrm{peak}}$th spectra are typically between 0.95 and 1.05 times the corresponding threshold). PL spectra have been vertically stacked for the sake of clarity.

In order to obtain better evidence of the kind of information that ASE thresholds coming from different methods can provide, for each sample we have compared a PL spectrum at low excitation density as a reference for the spontaneous emission lineshape, the PL spectrum acquired at the excitation density closest to the lowest threshold, and the one closest to the $I_{\mathrm{peak}}$ ASE threshold (see Figure 5). We can immediately notice that for all the samples, the spectra corresponding to the minimum ASE threshold value only present a small variation of the spectral lineshape with respect to the spontaneous emission, which is due to weak ASE. In the case of the SRhB sample, the “low th” PL spectrum does not show evident differences from the “below th” one, and only a slight broadening is present; this evidences the high sensitivity of the spectral linewidth to the presence of ASE, allowing the detection of slight spectral variations, including even those not visually observable. On the contrary, all the spectra at the $I_{\mathrm{peak}}$ ASE threshold are characterized by an evident presence of the ASE band, indicating that this method allows the determination of an excitation regime in which the ASE gives a relevant contribution to the emission.

The choice of the method to be adopted to determine the ASE threshold is thus strictly related to the definition of the threshold itself, and to the kind of information that one wants to obtain. Since at low pump densities only the spontaneous emission exists, one possibility is to define the ASE threshold as the minimum excitation density that allows one to detect the amplification of the radiation. From this perspective, the best method is the one providing the lowest experimental value. On the other hand, from a practical point of view, it could be useful to determine the excitation regime at which the ASE is strong enough to start to dominate over the spontaneous emission. Concerning the first option, we found that the minimum ASE threshold values are obtained from the application of the visual and ${\mathrm{FWHM}}_{\mathrm{narr}}$ methods. Among them, the visual method is the most rapid to apply, as it does not need any data analysis, but only an experimental system allowing a fine control of the excitation density, in order to be more sensitive to spectral modifications.

The fact that the ${\mathrm{FWHM}}_{\mathrm{narr}}$ method often provides low ASE thresholds confirms the high sensitivity of the spectral linewidth to the presence of ASE, as previously observed in polymeric waveguides [35]. However, in light of the evidence that the FWHM can depend on the excitation density even when only spontaneous emission is present (see Figure 2f and Figure S2c) and that the ASE appearance could also result in an initial FHWM increase (see Figure 3c), we suggest a *FWHM deviation* (${\mathrm{FWHM}}_{\mathrm{dev}}$) method, searching for the lowest pump density that provides a variation with respect to the initial conditions; this can be a narrowing, a broadening, or a slope change in the FWHM plot.

On the other hand, the methods mostly spread in the literature that exploit the excitation density dependence of the output intensity or the FWHM/2 properly describe the dominance of the ASE process in the PL spectra. Among them, the method $I_{\mathrm{peak}}$ allows a reliable estimate in the simplest way.

As a last point, comparing the results obtained for the various materials, we observe that the relative difference between the lowest and the highest threshold coming from the application of different methods is more pronounced in dye-polymer blends (up to six times for SRhB) with respect to perovskite waveguides (typically comparable within 30%, with the exception of the quasi-2D sample), even if the dependence on the method is similar.

The strongly reduced extent of the variation among ASE thresholds in perovskite NCs and 3D films is ascribed to the higher efficiency of the ASE process compared to the dye-polymer samples. This results in a narrower excitation density range in which the transition from a regime of pure spontaneous emission to a regime of ASE dominance takes place, making the difference among the ASE thresholds obtained from different methods small. This attribution is also confirmed by the higher difference between the values obtained for the quasi-2D film, that is, the sample showing the smoother transition from spontaneous emission to ASE-dominated spectra.

## 4. Materials and Methods

### 4.1. Sample Preparation and Characterization

We selected two classes of active materials whose ASE properties had already been investigated [26,36,37,38,39,40]. Concerning the lead halide perovskite family, we analyzed fully inorganic ${\mathrm{CsPbBr}}_{3}$ NC thin films (deposited on a substrate with and without HMDS-functionalization) [36], a bulk polycrystalline thin film of a hybrid organic-inorganic ${\mathrm{MAPbBr}}_{3}$ perovskite [37], and a thin film of quasi-2D ${\mathrm{BA}}_{3}{\mathrm{MA}}_{3}{\mathrm{Pb}}_{5}{\mathrm{Br}}_{16}$ perovskite [26].

As dye-doped polymer samples, we chose three reference active molecules: SRhB [39], rosamine4 [40] (both embedded in pHEMA films), and PO [38] with PMMA as a polymer matrix.

**VPI measurements for perovskite samples (NC, NCsub, MAPB, and Quasi-2D)**

PL and ASE measurements on the films were performed using an LTB MNL 100 nitrogen laser (Lasertechnik Berlin, Berlin, Germany) as excitation source, delivering 3 ns pulses at 337 nm with a repetition rate of 10 Hz. The pump laser was focused by a cylindrical lens in order to obtain a rectangular stripe of 4 mm length and 80 μm width. The laser excitation density was varied by a variable neutral filter. The PL emission, which was waveguided by the active film, was collected from the sample edge at the corresponding end of the excitation stripe through an optical fiber coupled to an ACTON SpectraPro-750 spectrometer (Acton Research Corporation, Acton, MA, USA) equipped with an Andor Peltier cooled CCD (Oxford Instruments, Abington, UK). The spectral resolution was 0.5 nm.

**VPI measurements for dye-polymer samples (SRhB, PO and rosamine4)**

The thin film samples were optically pumped at 532 nm with 20 ns FWHM pulses from a frequency-doubled Q-switched Nd:YAG laser (Lotis TII SL-2132 - Tokyo Instruments, Tokyo, Japan), operated at a 15 Hz repetition rate; the pump radiation was vertically or horizontally polarized. The light incident on the sample was perpendicular to the film surface and focused in a stripe shape spot of 150 μm width and 2 mm length. The edge ASE emission was collected with a 5 cm focal length spherical lens focused onto a fiber bundle and detected with a spectrograph/monochromator (Spectrapro-300i, Acton Research Corporation, Acton, MA, USA) equipped with a thermoelectrically cooled CCD detector (SpectruMM:GS 128B, Acton Research Corporation, Acton, MA, USA). Neutral density filters were used to avoid CCD detector saturation.

A full description of the sample preparation and the ASE measurement set-ups can be found in the corresponding original papers [26,36,37,38,39,40].

### 4.2. Methods Used to Extract the ASE Threshold

For each sample, we determined the ASE threshold by investigating the excitation density dependence of the PL spectra lineshape, intensity, or linewidth. In particular:

1. Visual method

The threshold was determined by observing the PL spectrum lineshape when the excitation density is increased, considering as threshold the lowest excitation density value that allows the observation of a variation of the spectral line-shape, due to the ASE band appearance.

2. Slope variation in the output intensity plot-based methods

These methods consider the ASE threshold as the excitation density value at which the slope of increase of the spontaneous emission intensity with the excitation density grows due to the contribution of the ASE intensity.

The slope variation excitation density is obtained as the crossing point between a linear best-fit line of the initial intensity increase and a following linear increase, with higher slope where the intensity increase becomes stronger. The error bar on the crossing point is obtained by determining a best-fit line range, varying the intercept of the best-fit lines within one standard deviation from the best-fit value, and determining the excitation density range between the crossing points of the extreme best-fit lines.

In the $I_{\mathrm{TOT}}$ method, the procedure is applied to the plot of the total intensity, integrated across the sample emission range. In the $I_{\mathrm{ASE}}$ method, only the ASE integrated intensity is considered, after separating its contribution from the spectrally integrated spontaneous emission one, as described in Ref [35]. Finally, in the $I_{\mathrm{peak}}$ method, the intensity at the ASE peak wavelength is considered.

3. FWHM-based methods

These methods quantify the ASE threshold by analyzing the spectral FWHM dependence on the excitation density. As the ASE band is narrower than the spontaneous emission one, the ASE appearance typically leads to a line narrowing.

In order to have a quantitative description of this behavior, we performed a best fit with a constant or linearly decreasing function at low excitation density, a linear fit for the line narrowing excitation density range, and finally a best fit to a constant for high excitation densities. The intervals of the best-fit functions are determined by changing the constant term in the fit function within one standard deviation as obtained by the fitting procedure, and they are used to estimate the uncertainty of the threshold values.

Starting from the best-fit curves, the ASE threshold was determined with three different methods:

$FWHM_{dev}$*method*: starting from the first point that deviates for more than one standard deviation from the initial constant/linear best fit, the ASE threshold value is calculated as the average between the excitation density value of this point and the one immediately before; semidispersion is used as maximum error and converted to statistical error;
$FWHM_{cross}$*method*: the ASE threshold is given by the excitation density value of the crossing point between the initial constant/linear fit at low excitation densities and the following decreasing linear fit. The intersection of the error lines are used for the determination of the ASE threshold error;
*FWHM/2 method*: the ASE threshold is given by the excitation density at which the FWHM halves with respect to the initial value obtained at low excitation density (line-width of the spontaneous emission spectrum). In the samples showing a FWHM variation before the ASE-induced line narrowing, we considered as reference the spectral linewidth of the PL spectrum at the lowest excitation density.

## 5. Conclusions

In this work, we have quantitatively investigated the ASE threshold dependence on the method used to determine it for dye-doped polymeric and lead halide perovskite thin films.

We have analyzed the ASE properties of three dye-doped polymer and four lead halide perovskite thin films and quantitatively compared the ASE threshold values obtained with the application of the seven methods most spread in the literature. We have also demonstrated that the best way to determine the beginning of the ASE process is the determination of the lowest excitation density that allows the observation of a variation of the spontaneous emission line-shape. In addition, we have shown that the methods most spread in the literature based on the slope change in the intensity growth with the excitation density allow the determination of the excitation regime in which ASE becomes the dominant emission process. In particular, the slope change of the intensity at the ASE peak wavelength provided the best compromise between reliable threshold values and ease of application. Finally, we have demonstrated that the quantitative difference between the two kinds of ASE thresholds strongly depends on the strength of the ASE increase above the threshold, from about 30% in samples with efficient ASE (such as most of the investigated lead-halide perovskite films) to more than six times in samples with gradually increasing ASE.

Our results and their general validity for different classes of active materials clearly demonstrate that great care must be taken before using the ASE threshold values in comparisons between different active materials.

In order to allow a complete and correct characterization of the ASE properties of a novel material and a meaningful quantitative comparison with already existing materials, we strongly suggest determining the beginning of the ASE by applying the visual method and the dominance of ASE by applying the $I_{\mathrm{peak}}$ method.

## Figures and Tables

**Table 1:** ASE threshold values obtained from the application of all the methods for the perovskite samples.

	ASE Threshold ($\mathrm{mJ}\;{\mathrm{cm}}^{-2}$)			
	**NCsub**	**NC**	**MAPB**	**Quasi-2D**
Visual	∼0.62	∼0.75	∼0.22	∼0.61
$I_{\mathrm{TOT}}$	0.652±0.025	1.01±0.18	0.267±0.024	–
$I_{\mathrm{ASE}}$	0.643±0.019	0.884±0.081	0.254±0.009	0.842±0.053
$I_{\mathrm{peak}}$	0.651±0.030	0.931±0.078	0.252±0.006	1.165±0.093
${\mathrm{FWHM}}_{\mathrm{narr}}$	0.480±0.040	0.688±0.033	0.240±0.012	1.14±0.21
${\mathrm{FWHM}}_{\mathrm{cross}}$	0.614±0.074	0.827±0.032	0.2571±0.0004	0.788±0.031
FWHM/2	0.656±0.075	0.977±0.030	0.286±0.014	1.558±0.014

**Table 2:** ASE threshold values obtained from the application of all the methods for dye-polymer blend samples.

	ASE Threshold ($\mathrm{MW}\;{\mathrm{cm}}^{-2}$)		
	**SRhB**	**PO**	**Rosamine4**
Visual	∼0.075	∼0.078	∼0.62
$I_{\mathrm{TOT}}$	0.202±0.018	–	–
$I_{\mathrm{ASE}}$	0.1292±0.0056	0.0926±0.0083	0.946±0.047
$I_{\mathrm{peak}}$	0.149±0.010	0.127±0.012	1.062±0.081
${\mathrm{FWHM}}_{\mathrm{narr}/\mathrm{dev}}$	0.0337±0.0022	0.0691±0.0053	0.720±0.060
${\mathrm{FWHM}}_{\mathrm{cross}}$	0.1485±0.0068	0.077±0.010	0.777±0.058
FWHM/2	0.2128±0.0060	0.1021±0.0079	1.598±0.053

## Data Availability

The data presented in this study are available on request from the corresponding author.

## Supplementary Materials

The following are available online at https://www.mdpi.com/article/10.3390/molecules27134261/s1, Figure S1: Excitation density dependence of the PL spectra, output intensity, and FWHM of the quasi-2D perovskite sample; Figure S2: Excitation density dependence of the PL spectra, output intensity, and FWHM of the MAPB perovskite sample; Figure S3: Excitation density dependence of the PL spectra, output intensity, and FWHM of the rosamine4-pHEMA blend; Figure S4: Excitation density dependence of the PL spectra, output intensity, and FWHM of the PO-PMMA blend; Figure S5: Total integrated intensity plots for quasi-2D perovskite thin film, PO, and rosamine4 samples.
