# Supplementary material for: Amplified Spontaneous Emission Threshold Dependence on Determination Method in Dye-Doped Polymer and Lead Halide Perovskite Waveguides
Source: Molecules. 2022 Jul 1;27(13):4261. doi: 10.3390/molecules27134261 (PMC9268657; doi:10.3390/molecules27134261)
Supplement: Supplementary file 1 [file molecules-27-04261-s001.zip › molecules-1732676-supplementary.pdf]

Supplementary Information
Amplified Spontaneous Emission Threshold Dependence on Determination Method in Dye-Doped Polymer and Lead Halide Perovskite Waveguides

S. Milanese[1,*], M. L. De Giorgi[1], L. Cerdán[2], M. G. La-Placa[2], N. F. Jamaludin[3], A. Bruno[3], H. J. Bolink[2], M. V. Kovalenko[4,5], and M. Anni[1]

July 1, 2022

Perovskite samples

Quasi-2D $BA_3MA_3Pb_5Br_{16}$ perovskite

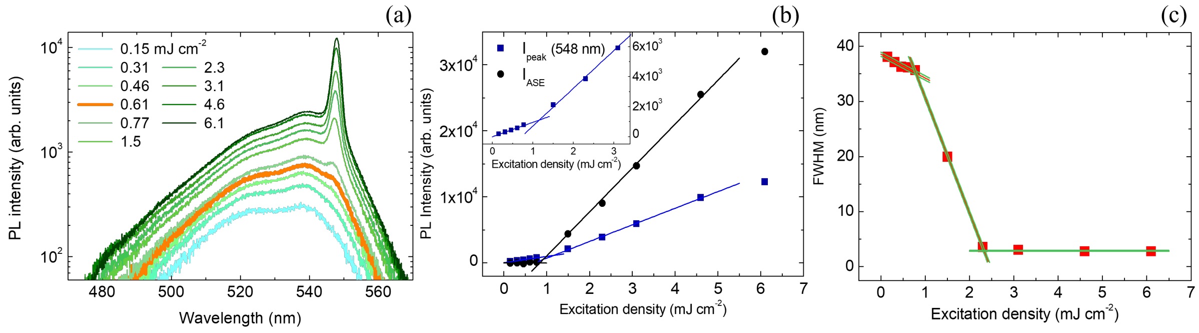

Figure S1: (a) Excitation density dependence of the PL spectra of the quasi-2D BAMAPbBr sample. The thicker orange line evidences the first spectrum in which the lineshape is modified by the ASE presence. (b) Excitation density dependence of the ASE integrated intensity and of the intensity at the ASE band peak wavelength. Inset: zoom of the I_{peak} plot corresponding to the slope change. (c) Excitation density dependence of the PL spectra FWHM.

Figure S1 summarizes the experimental results obtained for quasi-2D perovskite sample. In particular, Figure S1a shows the PL spectra as a function of the excitation density, from a minimum of 0.15 mJ cm^{-2} up to a maximum value of about 6 mJ cm^{-2}; it also evidences the first spectrum showing a slight modification with respect to the spontaneous emission spectral lineshape, resulting in the visual ASE threshold (0.61 mJ cm^{-2}). Figure S1b shows the excitation density dependence of the ASE integrated intensity and ASE peak intensity, which allows the determination of the relative ASE threshold values (0.842 ± 0.053) mJ cm^{-2} and (1.165 ± 0.093) mJ cm^{-2}. The excitation density dependence of the spectral linewidth is shown in Figure S1c; FWHM suffers a narrowing right from the lowest pump values $(0 - 1$ mJ cm$^{-2})$ before proceeding with a stronger decrease for higher excitation densities $(1 - 1.5$ mJ cm$^{-2})$ and finally a constant plateau $(2.5 - 6$ mJ cm$^{-2})$. The application of the FWHM-based methods results in the following ASE thresholds: (1.14 ± 0.21) mJ cm^{-2} ($FWHM_{narr}$), (0.788 ± 0.031) mJ cm^{-2} ($FWHM_{cross}$) and (1.558 ± 0.014) mJ cm^{-2} (FWHM/2).

$MAPbBr_3$ perovskite

The analysis of the ASE properties has been provided also for $MAPbBr_3$ perovskite thin film. Figure S2a shows some PL spectra at increasing excitation densities that highlight the appearance of the ASE process at about 0.2 mJ cm^{-2} (orange thicker line).
The study of the excitation density dependence of the output intensity (Figure S2b) allows the determination of the following ASE thresholds: (0.267 ± 0.024) mJ cm^{-2} (I_{TOT}), (0.254 ± 0.009) mJ cm^{-2} (I_{ASE}) and (0.252 ± 0.006) mJ cm^{-2} (I_{peak}). Starting from the analysis of the luminescence properties, we obtained the FWHM plot as a function of the excitation density (Figure S2c). A sudden narrowing has been observed

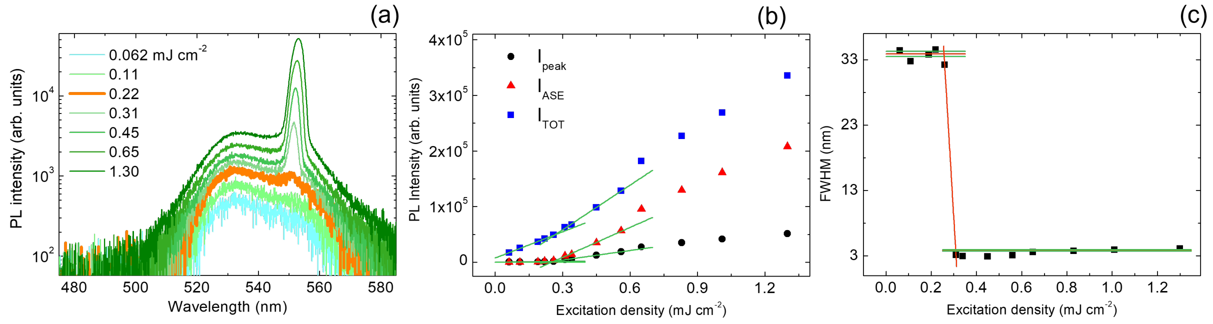

Figure S2: (a) Selection of the PL spectra of $MAPbBr_3$ perovskite sample at different excitation densities. The orange line evidences the first spectrum at which ASE starts to be visible. (b) Excitation density dependence of the Total integrated intensity, ASE integrated intensity and ASE peak intensity. (c) Plot of the FWHM as a function of the pump density.

at about 0.25 mJ cm^{-2} preventing the application of a fitting procedure and the determination of the uncertainty range for the decreasing part of the plot (0.25 − 0.3 mJ cm^{-2}). However, all the ASE thresholds have been extracted: (0.240 ± 0.012) mJ cm^{-2} for $FWHM_{narr}$, (0.2571 ± 0.0004) mJ cm^{-2} for $FWHM_{cross}$ and (0.286 ± 0.014) mJ cm^{-2} for FWHM/2 method.

Dye-polymer samples

Rosamine4-pHEMA blend

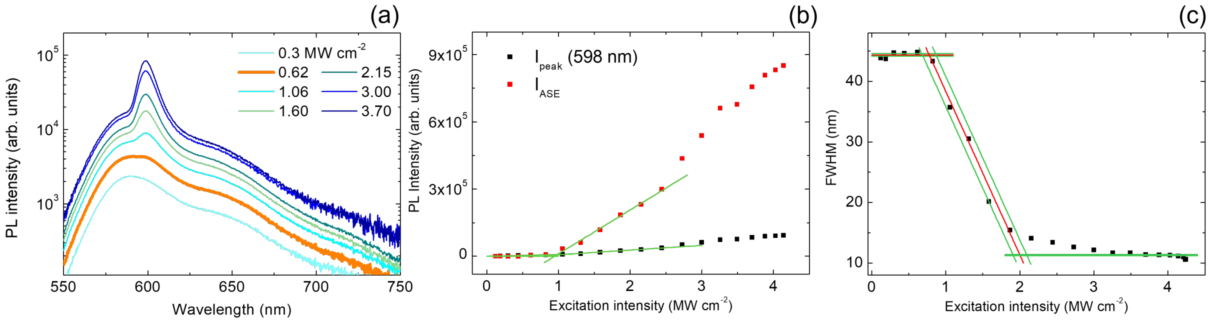

Figure S3: (a) Excitation intensity dependence for a selection of the PL spectra of the Rosamine4 sample. The thicker orange line evidences the first spectrum in which the lineshape is modified by the ASE presence.(b) Excitation intensity dependence of the ASE Integrated Intensity and ASE peak Intensity. (c) Excitation intensity dependence of the PL spectra FWHM.

In figure S3 we provide the analysis of the spectral features for Rosamine4 sample. Figure S3a reports a selection of the PL spectra acquired as a function of the input energy intensity: at low pump intensities (below 0.6 MW cm^{-2}) the emission spectrum is characterized by a broad band peaked at about 580 nm and a shoulder at about 640 nm. Once the excitation intensity increases, a small modification of the spectrum starts to be noticeable, and an ASE peak dominates at about 600 nm. The thicker orange line in Figure S3a evidences the first pump intensity at which the PL spectrum begins to deviate from the spontaneous emission lineshape and represents the visual ASE threshold (0.62 MW cm^{-2}).
The analysis of the excitation intensity dependence of the output intensity is provided in Figure S3b. The I_{TOT} plot of the Rosamine4 sample lacks the typical kink, preventing the determination of the ASE threshold. The ASE thresholds obtained from the application of the I_{ASE} and I_{peak} methods are (0.946 ± 0.047) MW cm^{-2} and (1.062 ± 0.081) MW cm^{-2}, respectively.
The analysis of the evolution of the spectral linewidth at increasing excitation intensity allowed to obtain the plot in Figure S3c in which the FWHM is shown. At low pump value (below 0.6 MW cm^{-2}) the width of the PL spectra remains constant around 45 nm; then for greater excitation intensities (0.7 − 2.0 MW cm^{-2}) it suffers a narrowing and finally reaches a minimum plateau for higher pump intensity centered at about 10 nm, sign of the fact that the ASE peak is dominating on the emission spectrum. The FWHM plot allows to extract the three ASE threshold values. The beginning of the line narrowing is set at (0.720 ± 0.060) MW cm^{-2}

($FWHM_{narr}$ method); the crossing of the two best fit lines corresponding to the knee of the curve provides a threshold of (0.777 ± 0.058) MW cm^{-2} ($FWHM_{cross}$), whereas the application of the FWHM/2 method results in (1.598 ± 0.053) MW cm^{-2}.

PO-PMMA blend

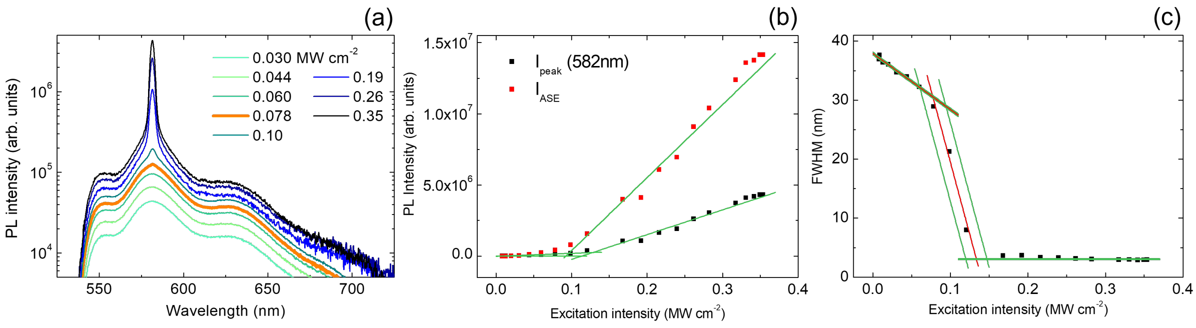

Figure S4: (a) Excitation intensity dependence of the PL spectra for the PO sample. (b) Excitation intensity dependence of the integrated ASE intensity (I_{ASE}) and of the intensity at the ASE band peak wavelength (I_{peak}).(c) Excitation intensity dependence of the PL spectra FWHM.

At low pump values, Perylene Orange spontaneous emission spectrum is characterized by a broad band with a central peak centered at about 580 nm and two shoulders at about 550 nm and 640 nm, respectively. The shape of the emission spectrum remains unchanged until an excitation intensity of about 0.078 MW cm^{-2} is provided, allowing us to extract the first ASE threshold value for the visual method and fix the ASE peak wavelength at 582 nm.
The excitation intensity dependence of the output intensity and the FWHM for PO are then introduced in Figure S4b and S4c, respectively. The absence of a slope variation in the excitation intensity dependence of the total emission prevents the determination the ASE threshold with the I_{TOT} method. The ASE thresholds obtained from the application of the I_{ASE} and I_{peak} methods are (0.0926 ± 0.0083) MW cm^{-2} and (0.127 ± 0.012) MW cm^{-2}, respectively.
Besides, looking at the excitation intensity dependence of the FWHM (Figure S4c), an immediate line narrowing is observed starting from the lowest excitation intensity values up to about 0.070 MW cm^{-2}, followed by a much stronger FWHM decrease for higher pump intensities (in the range $0.080 - 0.12$ MW cm^{-2}), up to the final minimum plateau for excitation intensity greater than 0.20 MW cm^{-2}. For this reason, similarly to what we did for the NC sample, we substituted the initial constant fit with a linear one, leaving unaltered the remaining fitting procedure. The application of the $FWHM_{narr}$, $FWHM_{cross}$ and FWHM/2 results in (0.0691 ± 0.0053) MW cm^{-2}, (0.077 ± 0.010) MW cm^{-2} and (0.1021 ± 0.0079) MW cm^{-2} as ASE threshold values, respectively.

Total Integrated Intensity plots

Figure S5 provides the plot of the output intensity integrated over the entire spectral range for the following samples: quasi-2D perovskite, PO and Rosamine4 dyes. None of them are characterized by the typical kink that allows the determination of the ASE threshold as the excitation density corresponding to the slope change.

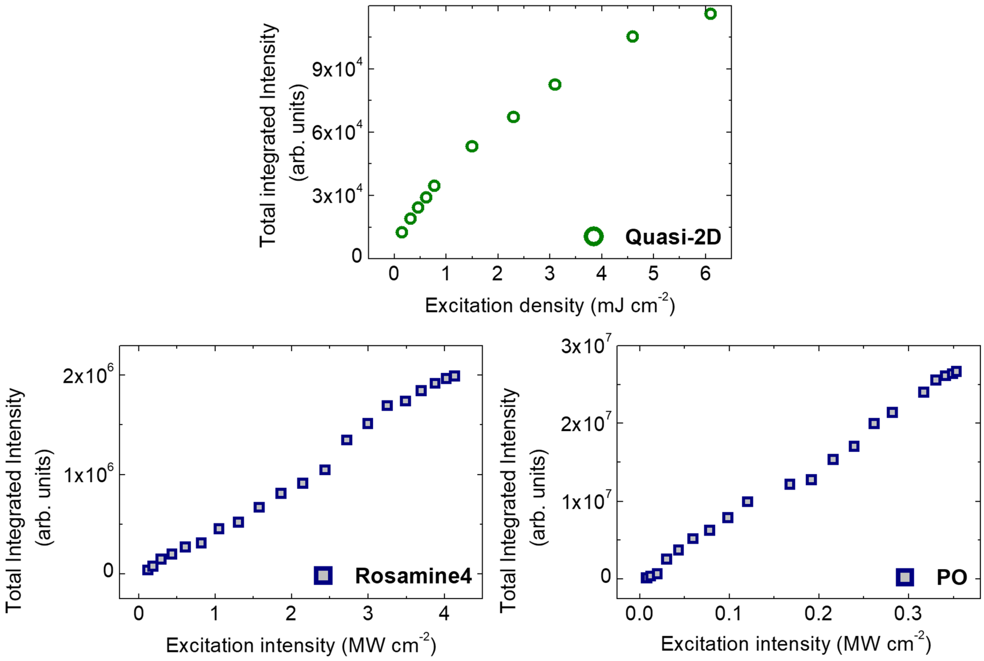

Figure S5: Total Integrated Intensity plot for Quasi-2D perovskite thin film (upper row), and Perylene Orange and Rosamine4 samples (bottom row).
